# Detection and discrimination of five *E. coli* pathotypes using a combinatory SYBR® Green qPCR screening system

**DOI:** 10.1007/s00253-018-8820-0

**Published:** 2018-02-20

**Authors:** Elodie Barbau-Piednoir, Sarah Denayer, Nadine Botteldoorn, Katelijne Dierick, Sigrid C. J. De Keersmaecker, Nancy H. Roosens

**Affiliations:** 1 Scientific Service Foodborne Pathogens, Scientific Institute of Public Health, J. Wytsmanstraat 14, B-1050 Brussels, Belgium; 2 Platform Biotechnology and Bioinformatics, Scientific Institute of Public Health, J. Wytsmanstraat 14, B-1050 Brussels, Belgium

**Keywords:** Real-time PCR, SYBR® Green, Detection, Pathogenic *E. coli*, STEC, Validation

## Abstract

A detection and discrimination system for five *Escherichia coli* pathotypes, based on a combination of 13 SYBR® Green qPCR, has been developed, i.e., combinatory SYBR® Green qPCR screening system for pathogenic *E. coli* (CoSYPS Path *E. coli*). It allows the discrimination on isolates and the screening of potential presence in food of the following pathotypes of *E. coli:* shigatoxigenic (STEC) (including enterohemorrhagic (EHEC)), enteropathogenic (EPEC), enteroaggregative (EAggEC), enteroaggregative shigatoxigenic (EAggSTEC), and enteroinvasive (EIEC) *E. coli*. The SYBR® Green qPCR assays target the *uidA*, *ipaH*, *eae*, *aggR*, *aaiC*, *stx1*, and *stx2* genes. *uidA* controls for *E. coli* presence and all the other genes are specific targets of *E. coli* pathotypes. For each gene, two primer pairs have been designed to guarantee a sufficient detection even in case of deletion or polymorphisms in the target gene. Moreover, all the qPCR have been designed to be run together in a single analytical PCR plate. This study includes the primer pairs’ design, in silico and in situ selectivity, sensitivity, repeatability, and reproducibility evaluation of the 13 SYBR® Green qPCR assays. Each target displayed a selectivity of 100%. The limit of detection of the 13 assays is between 1 and 10 genomic copies. Their repeatability and reproducibility comply with the European requirements. As a preliminary feasibility study on food, the CoSYPS Path *E. coli* system was subsequently evaluated on four food matrices artificially contaminated with pathogenic *E. coli.* It allowed the detection of an initial contamination level as low as 2 to 7 cfu of STEC/25 g of food matrix after 24 h of enrichment.

**Electronic supplementary material:**

The online version of this article (10.1007/s00253-018-8820-0) contains supplementary material, which is available to authorized users.

## Introduction

*Escherichia coli* strains are enteric bacteria. Most of them are part of the beneficial natural flora of the human and animal intestine. Some strains have acquired mechanisms to cause diseases and have thus become pathogenic for humans. The *E. coli* strains are classified by their heat-stable somatic (O) antigens and flagellar (H) antigens, two major antigens of *Enterobacteriacea* (Ryan 2004), into >380 serotypes (Karmali et al. 2010). However, this classification gives no immediate information about the pathogenicity of the *E. coli* strain. Pathogenic *E. coli* are divided in two major groups: diarrhoeagenic *E. coli* (DEC) and extraintestinal *E. coli* (ExPEC)*.* These groups can be further categorized into pathotypes, based on the type of virulence factor present in the *E. coli* genome and based on the host clinical symptoms. Two pathotypes belong to the ExPEC, namely, uropathogenic (UPEC) and neonatal meningitidis (NMEC) *E. coli.* The DEC group consists of eight pathotypes, namely, shigatoxigenic (STEC) (including the enterohemorrhagic (EHEC) *E. coli* (Croxen and Finlay 2010; Croxen et al. 2013)), enteropathogenic (EPEC), enterotoxigenic (ETEC), enteroinvasive (EIEC; including *Shigella* spp.), enteroaggregative (EAggEC), diffusively adherent (DAEC), adherent invasive (AIEC), and the recently described enteroaggregative shigatoxigenic (EAggSTEC) *E. coli* (Clements et al. 2012; Croxen and Finlay 2010; Croxen et al. 2013). In the case of STEC, Karmali’s seropathotype classification is based on a serotype-specific spectrum of disease frequency and severity, since only a limited number of serotypes appear to be associated with the majority of human disease (Karmali et al. 2010).

The natural reservoirs of pathogenic *E. coli* are the intestinal tracts of animals, mainly ruminants (Clements et al. 2012). Human infection occurs mainly by consumption of contaminated food products of animal origin, contaminated raw food products such as salads, drinking water contaminated with animal or human waste, or through direct person-to-person spread due to poor hygiene but also through direct contact with infected animals (Clements et al. 2012; Karmali et al. 2010). The STEC pathotype causes mostly sporadic disease and is the most important in terms of number of human cases (European Food Safety Authority (EFSA), European Centre for Disease Prevention and Control (ECDC) 2013, 2015, European Food Safety Authority (EFSA), European Centre for Disease Prevention and Control (ECDC) Prevention and Control 2016). STEC’s (or VTEC) pathogenicity is linked to the production of shiga-toxins (sometimes referred to as Vero toxins) (encoded by *stx1* and/or *stx2*) in combination with an epithelial cell adhesion factor (encoded by the intimin gene (*eae*)) (Clements et al. 2012; Karmali et al. 2010). STEC can cause mild to bloody diarrhea often with abdominal cramps and fever. Hemolytic-ureamic syndrome (HUS) is a severe complication of STEC and can lead to acute renal failure in young children (EFSA and ECDC 2013, 2015, European Food Safety Authority (EFSA), European Centre for Disease Prevention and Control (ECDC) Prevention and Control 2016). In the European Union (EU), STEC is the fourth cause of human zoonosis with 5995 confirmed human cases in 2014, a hospitalization rate of 39.2% and a fatality rate of 0.20% (European Food Safety Authority (EFSA), European Centre for Disease Prevention and Control (ECDC) Prevention and Control 2016). In 2011, these statistics were even worse due to a large outbreak caused by sprouts contaminated with an O104:H4 serotype of *E. coli* (EAggSTEC) in Germany and France (ECDC 2013; King et al. 2012; Wadl et al. 2011). The strain involved in this large outbreak had an unusual combination of pathogenic features typical for enteroaggregative *E. coli* (EAggEC) together with the capacity to produce shiga-toxin 2 (*stx2*), the latter being the hallmark of STEC (Scheutz et al. 2011). Consequently, the EU extended the regulation 2073/2005 on the microbiological criteria for foodstuffs (Commission of the European Communities 2005) with the amendment 209/2013 (Commission of the European Communities 2013). These adaptations propose significant changes in the control strategy, especially to test for the presence of the top five STEC serogroups causing human disease (i.e., O157, O26, O111, O103, and O145) and the O104:H4 serotype involved in the German outbreak.

To comply with this new EU legislation, a new standard for the detection of these serogroups was published, i.e., the ISO/TS 13136:2012 (ISO: International Organization for Standardization 2012), and includes a qPCR detection assay targeting the *stx*-genes as a first screening for STEC (ISO: International Organization for Standardization 2012) prior to detection of the serogroup. Several other qPCR assays for *stx* and other virulence genes are available in literature but most of them are limited to STEC detection (e.g., Anklam et al. 2012; Bugarel et al. 2010; Nielsen and Andersen 2003; Paton and Paton 1998; Pavlovic et al. 2010; Perelle et al. 2004; Sharma and Dean-Nystrom 2003; Wasilenko et al. 2014). Moreover, some methods allowing the detection of several pathotypes of *E. coli* such as STEC, EAggEC, EHEC*,* EPEC, ETEC, EIEC, and DAEC, targeting other virulence genes, have been developed (e.g., multiplex conventional PCR (Aranda et al. 2004; Baccin Fialho et al. 2013; Botteldoorn et al. 2003; Chandra et al. 2013; Kuwayama et al. 2011) and more recently also qPCR assays (Barletta et al. 2013; Fukushima et al. 2009; Liu et al. 2013; Tzschoppe et al. 2012)). However, these detection systems are not compatible to run under the same condition and target a single gene within a single assay. This leads to a diagnosis based on multiple experiments performed on different analytical runs and can lead to false negative results in case of gene mutation or deletion in the annealing site of the primers of the targeted genes (Barbau-Piednoir et al. 2013b).

In this paper, we report the development of the pathogenic *E. coli* detection system (CoSYPS Path *E. coli*) based on a set of 13 qPCR assays targeting 7 genes of interest that can be combined to be used in a single run in a 96-well plate format. Two assays target most of *E. coli* (*uidA* gene) and the 11 other assays target 6 different markers of 5 *E. coli* pathotypes with two assays per target. Applied to isolates, this detection system allows the discrimination of five *E. coli* pathotypes, i.e., STEC (including EHEC), EPEC, EAggEC, EAggSTEC, and EIEC.

In addition, this CoSYPS Path *E. coli* would be of great interest for food matrices screening as it replies to the need of detecting virulence factors from *E. coli* pathotypes other than those from STEC, i.e., EAggEC, EAggSTEC, EIEC, and EPEC. This CoSYPS Path *E. coli* would be an appropriate tool to better evaluate by screening the possible presence of a broader range of pathogenic *E. coli* in a food sample.

## Materials and methods

### Bacterial strains

The bacterial isolates used in this study are listed in Table 1. A panel of DNA extracted from 113 bacterial isolates (76 *E. coli* isolates, 3 others species of the *Escherichia* genus, and 34 isolates from 22 other genera), two mold species and three animal species has been tested. The bacterial isolates were obtained from National Reference Centres and Laboratories. The meat samples were purchased at a retail shop.

**Table 1:** Selectivity assessment of the 13 SYBR® Green qPCR assays of the CoSYPS Path *E. coli*

				*E. coli* markers		EIEC (and *Shigella*) markers		*eae* presence	EPEC and typical EHEC markers		*stx* presence and type	STEC and EAggSTEC markers			EAggEC and EAggSTEC markers			
	Bacteria name	Reference	Strain provider	*uidA*-3	*uidA*-7	*ipaH*-569	*ipaH*-3		*eae*-185	*eae*-EBP-1		*stx1*and*2*–4	*stx1*–185	*stx2*–81-alt	*aggR*-185	*aggR*-2	*aaiC*-EBP1-deg	*aaiC*-EBP2-tronc
STEC (including EHEC)	*E. coli* O118:H16	EH1641	5	+	+	–	–	+	+	+	+, *stx1a*	+	+	–	–	–	–	–
	*E. coli* O118:H16	EH1766	5	+	+	–	–	+	+	+	+, stx1a	+	+	–	nt	nt	nt	nt
	*E. coli* O145:H-	EH1492	5	+	+	–	–	+	+	+	+, *stx1*	+	+	–	–	–	–	–
	*E. coli* O103	EH1717	5	+	+	–	–	+	+	+	+, *stx1*	+	+	nt	–	–	–	–
	*E. coli* O111:H-	EH 1999	5	+	+	–	–	+	+	+	+, *stx1*	+	+	nt	–	–	–	–
	*E. coli* O145	TIAC 779	3	+	+	–	–	+	+	+	+, *stx1*	+	+	–	nt	nt	nt	nt
	*E. coli* O145	TIAC 783	3	+	+	–	–	+	+	+	+, *stx1*	+	+	–	nt	nt	nt	nt
	*E. coli* O145:H-	EH1992	5	+	+	–	–	+	+	+	+, *stx1*	+	+	–	nt	nt	nt	nt
	*E. coli* O145:H-	EH1995	5	+	+	–	–	+	+	+	+, *stx1*	+	+	nt	nt	nt	nt	nt
	*E. coli* O26	EH1823	5	+	+	–	–	+	+	+	+, *stx1*	+	+	–	nt	nt	nt	nt
	*E. coli* O26	EH1839	5	+	+	–	–	+	+	+	+, *stx1*	+	+	nt	nt	nt	nt	nt
	*E. coli*	MB2651 - TIAC 1747	2–3	+	+	–	–	+	+	+	+, *stx1*	+	+	nt	–	–	–	–
	*E. coli*	MB 2668 - TIAC 1750	2–3	+	+	–	–	+	+	+	+, *stx1*	+	+	nt	–	–	–	–
	*E. coli*	MB 3068 - TIAC 1763	2–3	+	+	–	–	+	+	+	+, *stx1*	+	+	nt	nt	nt	nt	nt
	*E. coli*	MB 2680 - TIAC 1753	2–3	+	+	–	–	+	+	+	+, *stx1*	+	+	–	–	–	–	–
	*E. coli*	MB 2681 - TIAC 1754	2–3	+	+	–	–	+	+	+	+, *stx1*	+	+	–	–	–	–	–
	*E. coli*	MB2777 - TIAC 1757	2–3	+	+	–	–	+	+	+	+, *stx1*	+	+	–	nt	nt	nt	nt
	*E. coli* O157:H-	EH1373	5	+	+	–	–	+	+	+	+, *stx1*-*stx2c*	+	+	+	nt	nt	nt	nt
	*E. coli* O55:H7	EH 2127	5	+	+	–	–	+	+	+	+, *stx1*-*stx2a*	+	+	+	nt	nt	nt	nt
	*E. coli* O26	TIAC 778	3	+	+	–	–	+	+	+	+, *stx1*-*stx2*	+	+	+	nt	nt	nt	nt
	*E. coli* O111	EH1811	5	+	+	–	–	+	+	+	+, *stx1*-*stx2*	+	+	+	–	–	–	–
	*E. coli* O157:H7	EH899	5	+	+	–	–	+	+	+	+, *stx1-stx2*	+	+	+	nt	nt	nt	nt
	*E. coli* O157:H7	EH1829	5	+	+	–	–	+	+	+	+, *stx1*-*stx2*	+	+	+	nt	nt	nt	nt
	*E. coli* O157:H7	EH1230	5	+	+	–	–	+	+	+	+, *stx1*-*stx2*	+	+	+	–	–	–	–
	*E. coli* O157:H7	EH1229	5	+	+	nt	nt	+	+	+	+, *stx1*-*stx2*	+	+	+	nt	nt	nt	nt
	*E. coli* O154	TIAC 935	3	+	+	–	–	+	+	+	+, *stx1*-*stx2*	+	+	+	nt	nt	nt	nt
	*E. coli* O157:H7	TIAC 924	3	+	+	nt	nt	+	+	+	+, *stx1a*-*stx2a*	+	+	+	nt	nt	nt	nt
	*E. coli* O182:H25	EH2038	5	+	+	–	–	+	+	+	+, *stx1a*- *stx2*	+	+	+	nt	nt	nt	nt
	*E. coli* O103	TIAC 776	3	+	+	nt	nt	+	+	+	+, *stx2*	+	–	+	nt	nt	nt	nt
	*E. coli* O121	TIAC 780	3	+	+	nt	nt	+	+	+	+, *stx2*	+	–	+	nt	nt	nt	nt
	*E. coli* O145	EH1846	5	+	+	nt	nt	+	+	+	+, *stx2*	+	–	+	–	–	–	–
	*E. coli* O157:H7	EH1819	5	+	+	nt	nt	+	+	+	+, *stx2*	+	–	+	nt	nt	nt	nt
	*E. coli* O157:H7	EH845	5	+	+	nt	nt	+	+	+	+, *stx2*	+	–	+	nt	nt	nt	nt
	*E. coli* O157:H7	EH851	5	+	+	nt	nt	+	+	+	+, *stx2*	+	–	+	nt	nt	nt	nt
	*E. coli* O157:H7	EH1084	5	+	+	nt	nt	+	+	+	+, *stx2*	+	–	+	–	–	–	–
	*E. coli* O145:H28	EH1533	5	+	+	nt	nt	+	+	+	+, *stx2*	+	–	+	nt	nt	nt	nt
	*E. coli* O157:H7	EH1237	5	+	+	nt	nt	+	+	+	+, *stx2*	+	–	+	nt	nt	nt	nt
	*E. coli* O84:H-	EH1861	5	+	+	–	–	+	+	+	‘+, *stx2a*	+	–	+	nt	nt	nt	nt
	*E. coli* O157:H7	EH1310	5	+	+	–	–	+	+	+	+, *stx2c*	+	–	+	–	–	–	–
	*E. coli* O157:H7	EH1344	5	+	+	–	–	+	+	+	+, *stx2c*	+	–	+	nt	nt	nt	nt
	*E. coli* O128	TIAC 929	3	+	+	–	–	+	+	+	+, *stx2f*	–	–	+	nt	nt	nt	nt
	*E. coli* O128ac:H-	EH1666	5	+	+	–	–	+	+	+	+, *stx2f*	–	–	+	nt	nt	nt	nt
	*E. coli* O63:H6	EH1836	5	+	+	–	–	+	+	+	+, *stx2f*	–	–	+	nt	nt	nt	nt
	*E. coli* O63:H6	EH1882	5	+	+	–	–	+	+	+	+, *stx2f*	–	–	+	nt	nt	nt	nt
	*E. coli*	MB3038 - TIAC 1762	2–3	+	+	–	–	+	+	+	+, *stx2f*	–	–	+	nt	nt	nt	nt
	*E. coli*	MB4379 - TIAC 1764	2–3	+	+	–	–	+	+	+	+, *stx2f*	–	–	+	nt	nt	nt	nt
	*E. coli* O5:H-	EH1648	5	+	+	–	–	–	nt	nt	+, *stx1c*	+	+	–	–	–	–	–
	*E. coli* O145	MB 3882	2	+	+	–	–	–	nt	nt	+, *stx1c*	+	+	–	nt	nt	nt	nt
	*E. coli* O8	TIAC 928	3	+	+	–	–	–	–	–	+, *stx1d*	+	+	–	nt	nt	nt	nt
	*E. coli* OX183:H18	EH1671	5	+	+	–	–	–	–	–	+, *stx1a*-*stx2a*	+	+	+	nt	nt	nt	nt
	*E. coli* O105:H18	EH1653	5	+	+	–	–	–	–	–	+, *stx1a*-*stx2a*	+	+	+	nt	nt	nt	nt
	*E. coli* O48	TIAC 923	3	+	+	–	–	–	–	–	+, *stx1* &*stx2a*	+	+	+	nt	nt	nt	nt
	*E. coli*	MB1448 - TIAC 1719	2–3	+	+	–	–	–	–	–	+, *stx2*	+	–	+	nt	nt	nt	nt
	*E. coli* O103	TIAC 933	3	+	+	–	–	–	–	–	+, *stx2*	+	–	+	–	–	–	–
	*E. coli* O118	TIAC 927	3	+	+	–	–	–	–	–	+, *stx2b*	+	–	+	nt	nt	nt	nt
	*E. coli* O91:H-	EH1771	5	+	+	–	–	–	–	–	+, *stx2b*	+	–	+	nt	nt	nt	nt
	*E. coli* O118:H12	EH0101	5	+	+	–	–	–	–	–	+, *stx2d*	+	–	+	nt	nt	nt	nt
	*E. coli* O118:H12	EH0250	5	+	+	–	–	–	–	–	+, *stx2d*	+	–	+	nt	nt	nt	nt
	*E. coli* O118:H12	EH0251	5	+	+	–	–	–	–	–	+, *stx2d*	+	–	+	nt	nt	nt	nt
	*E. coli* O8:H9	EH1785	5	+	+	–	–	–	–	–	+, *stx2e*	+	–	+	nt	nt	nt	nt
	*E. coli* O8:H-	EH1923	5	+	+	–	–	–	–	–	+, *stx2e*	+	–	+	nt	nt	nt	nt
	*E. coli* O2	TIAC 931	3	+	+	–	–	–	–	–	+, *stx2g*	+	–	+	nt	nt	nt	nt
EPEC	*E. coli* O111	TIAC 775	3	nt	nt	nt	nt	+	+	+	–	–	–	–	–	–	–	–
	*E. coli* O111	TIAC 784	3	nt	nt	nt	nt	+	+	+	–	–	–	–	–	–	–	–
	*E. coli* O157:H7	EH2045	5	nt	nt	nt	nt	+	+	+	–	–	–	–	nt	nt	nt	nt
	*E. coli* O157:H7	EH2046	5	nt	nt	nt	nt	+	+	+	–	–	–	–	nt	nt	nt	nt
	*E. coli* O157:H7	EH630	5	+	+	–	–	+	+	+	–	–	–	–	nt	nt	nt	nt
	*E. coli* O157:H7	EH857	5	+	+	nt	nt	+	+	+	–	–	–	–	nt	nt	nt	nt
	*E. coli* O157:H7	DIV5139	5	nt	nt	nt	nt	+	+	+	–	–	–	–	nt	nt	nt	nt
EAggSTEC	*E. coli* O104:H2	TIAC 944	3	+	+	–	–	–	–	–	+, *stx2a+*	+	–	+	+	+	+	+
	*E. coli* O104	TIAC 1951	3	+	+	–	–	–	–	–	+, *stx2a+*	+	–	+	+	+	+	+
	*E. coli* O104:H4	TIAC 2003	3	+	+	–	–	–	–	–	+, *stx2a+*	+	–	+	+	+	+	+
EAggEC	*E. coli* O104:H2	TIAC 2322	3	+	+	–	–	–	–	–	–	–	–	–	+	+	+	+
ETEC	*E. coli*	MB1441 - TIAC 1712	2–3	+	+	–	–	–	–	–	–	–	–	–	–	–	–	–
	*E. coli*	MB1540 - TIAC 1728	2–3	+	+	–	–	–	–	–	–	–	–	–	–	–	–	–
*E. coli*	*E. coli*	ATCC 25922	3	+	+	–	–	–	–	–	–	–	–	–	–	–	–	–
Other species of the genus *Escherichia*	*Escherichia fergusonii*	TIAC 674	3	–	–	–	–	–	–	–	–	–	–	–	–	–	–	–
	*Escherichia hermanii*	TIAC 668	3	–	–	–	–	–	–	–	–	–	–	–	–	–	–	–
	*Escherichia vulneris*	TIAC 675	3	–	–	–	–	–	–	–	–	–	–	–	–	–	–	–
*Shigella*	*Shigella boydii 2*	12–531	1	+	+	+	+	–	nt	nt	–	–	–	–	–	–	–	–
	*Shigella boydii 10*	12–2582	1	+	+	+	+	–	nt	nt	–	–	–	–	–	–	–	–
	*Shigella boydii 18*	12–3346	1	+	+	+	+	–	nt	nt	–	–	–	–	–	–	–	–
	*Shigella dysenteriae 2*	12–4544	1	–	–	+	+	–	–	–	–	–	–	–	–	–	–	–
	*Shigella dysenteriae 3*	12–1388	1	+	+	+	+	–	nt	nt	–	–	–	–	–	–	–	–
	*Shigella dysenteriae 4*	11–3617	1	–	–	+	+	–	–	–	–	–	–	–	–	–	–	–
	*Shigella dysenteriae 6*	10–1857	1	+	+	+	+	–	–	–	–	–	–	–	–	–	–	–
	*Shigella flexneri 2a*	*Oct-91*	1	+	+	+	+	–	–	–	–	–	–	–	–	–	–	–
	Shigella flexneri 2a	12–0081	1	+	+	+	+	–	nt	nt	–	–	–	–	–	–	–	–
	*Shigella flexneri 3b*	*12–0685*	1	+	+	+	+	–	nt	nt	–	–	–	–	–	–	–	–
	*Shigella flexneri y*	*12–1386*	1	+	+	+	+	–	nt	nt	–	–	–	–	–	–	–	–
	*Shigella sonneï*	*10–3865*	1	+	+	+	+	–	–	–	–	–	–	–	–	–	–	–
Other genera	*Aeromonas hydrophila*	M/2862	*4*	–	–	–	–	–	–	–	–	–	–	–	–	–	–	–
	*Bacillus cereus*	ATCC 14579	*3*	–	–	–	–	–	–	–	–	–	–	–	–	–	–	–
	*Brevibacillus bortelensis*	TIAC 099	*3*	–	–	–	–		–	–		–	–	–	–	–	–	–
	*Campylobacter jejuni*	ATCC 33291	*3*	–	–	–	–	–	–	–	–	–	–	–	–	–	–	–
	*Citrobacter freundii*	TIAC 554	*3*	–	–	–	–	–	–	–	–	–	–	–	–	–	–	–
	*Enterobacter aerogenes*	M/3785	*4*	–	–	–	–	–	–	–	–	–	–	–	–	–	–	–
	*Enterococcus faecalis*	ATCC 29212	*4*	–	–	–	–		–	–		–	–	–	–	–	–	–
	*Hafnia alvei*	*7186*	*4*	–	–	–	–	–	–	–	–	–	–	–	–	–	–	–
	*Klebsiella pneumoniae*	TIAC 446	*3*	–	–	–	–	–	–	–	–	–	–	–	–	–	–	–
	*Lactobacillus helveticus*	Argentijns vlees A12	*3*	–	–	–	–	–	–	–	–	–	–	–	–	–	–	–
	*Listeria monocytogenes*	ATCC 51772	*6*	–	–	–	–	–	–	–	–	–	–	–	–	–	–	–
	*Proteus mirabilis*	TIAC 726	*3*	–	–	–	–	–	–	–	–	–	–	–	–	–	–	–
	*Providencia rettgeri*	M/831	*4*	–	–	–	–	–	–	–	–	–	–	–	–	–	–	–
	*Pseudomonas aeruginosa*	LMG 6395	*3*	–	–	–	–	–	–	–	–	–	–	–	–	–	–	–
	*S. enterica enterica* Enteritidis	H, VI, 6, 32	1	–	–	–	–	–	–	–	–	–	–	–	–	–	–	–
	*S.enterica enterica* Thyphimurium	H, II, 32, 32	1	–	–	–	–	–	–	–	–	–	–	–	–	–	–	–
	*Serratia marcescens*	*7015*	*4*	–	–	–	–	–	–	–	–	–	–	–	–	–	–	–
	*Staphylococcus epidermidis*	TIAC 367	*3*	–	–													–
	*Staphylococcus aureus*	ATCC 25923	*3*	–	–	–	–	–	–	–	–	–	–	–	–	–	–	–
	*Streptococcus pyrogenes*	ATCC 19615	*3*	–	–	–	–	–	–	–	–	–	–	–	–	–	–	–
	*Vibrio parahaemolyticus*	TIAC 610	*3*	–	–	–	–	–	–	–	–	–	–	–	–	–	–	–
	*Yersinia enterocolitica*	LMG 15558	*3*	–	–	–	–	–	–	–	–	–	–	–	–	–	–	–
	Chicken: *Gallus gallus domesticus*	n.a.	n.a.	–	–	–	–	–	–	–	–	–	–	–	–	–	–	–
	Beef: *Bos primigenius taurus taurus*	n.a.	n.a.	–	–	–	–	–	–	–	–	–	–	–	–	–	–	–
	Pork: *Sus scrofa domesticus*	n.a.	n.a.	–	–	–	–	–	–	–	–	–	–	–	–	–	–	–
	*Candida parapsilosis*	BCCM/IHEM 6478	*7*	–	–	–	–	–	–	–	–	–	–	–	–	–	–	–
	*Cladosporium sphaerospermum*	BCCM/IHEM 24474	*7*	–	–	–	–	–	–	–	–	–	–	–	–	–	–	–
	*NTC*			–	–	–	–	–	–	–	–	–	–	–	–	–	–	–
	True positives			81	81	12	12		53	53		59	34	45	4	4	4	4
	False negatives			2	2	0	0		0	0		6	0	0	0	0	0	0
	True negatives			31	31	90	90		57	57		54	85	67	63	63	63	63
	False positives			0	0	0	0		0	0		0	0	0	0	0	0	0
	Total number of samples			114	114	102	102		110	110		119	119	112	67	67	67	67
	Inclusivity: sensibility (SE)			**97.6**	**97.6**	**100.0**	**100.0**		**100**	**100**		**90.8**	**100**	**100**	**100**	**100**	**100**	**100**
	Exclusivity: specificity (SP)			**100**	**100**	**100**	**100**		**100**	**100**		**100**	**100**	**100**	**100**	**100**	**100**	**100**
	Accuracy (AC)			**98.2**	**98.2**	**100.0**	**100.0**		**100**	**100**		**95**	**100**	**100**	**100**	**100**	**100**	**100**

### Bacterial growth conditions, DNA extraction, and DNA quantification

Overnight cultures of each bacterial isolate were grown in brain-heart infusion (BHI) broth or Bolton broth (for *Campylobacter*) at the appropriate temperature and oxygen condition. The total DNA from each of the bacterial isolates was extracted using the “Gram-negative or Gram-positive bacteria” protocol of the DNeasy Blood and Tissue Kit (Qiagen, Hilden, Germany). Fungal genomic DNA (gDNA) was extracted with the ZR Fungal/Bacterial gDNA Extraction Kit (Zymo Research, Irvine, CA, USA). The total DNA from meat was extracted using the “Animal Tissue” protocol of the DNeasy Blood and Tissue Kit (Qiagen, Hilden, Germany). All kits were used according to the manufacturer’s recommendations. The DNA quality was verified on agarose gel (1%) and the DNA concentration was measured using a NanoDrop® 2000 device (ThermoFisher Scientific, Schwerte, Germany).

### Calculation of bacterial genomic copy number

The bacterial genomic copy number was calculated according to the genome size of each targeted bacterial isolate using the formula published in Barbau-Piednoir et al. (2013a).

### Design and in silico assessment of primer pairs

A uniform primer design approach was applied in the development of all primer pairs, as previously described for the primer design for the *Salmonella* and *Listeria* detection and discrimination system (Barbau-Piednoir et al. 2013a, 2013b). The first step consisted of identifying genes of interest, either genus or pathotype specific, by means of a bibliographic study (Clements et al. 2012; Croxen and Finlay 2010). The second step included the collection of primer sequences available in the literature targeting the selected genes giving an amplicon between 60 and 120 bp (Anklam et al. 2012; Botteldoorn et al. 2003; Fukushima et al. 2009; Kim et al. 2010; Nielsen and Andersen 2003; Pavlovic et al. 2010; Perelle et al. 2004; Sharma et al. 1999; Takahashi et al. 2009; Thiem et al. 2004; Tzschoppe et al. 2012). If none were found, primer pairs were designed, preferentially within conserved regions, using the “Primer 3” program (http://frodo.wi.mit.edu/primer3/ (Rozen and Skaletsky 2000)) with the “product size range” specification set at “60 to 120 bp” and “primer size” optimal set at “22 bases.” In the third step, a collection of bacterial DNA sequences of other foodborne pathogenic bacteria and bacteria naturally present in food matrices was retrieved from the NCBI public database (http://www.ncbi.nlm.nih.gov/sites/entrez). An in silico test of the primer pairs was subsequently performed as previously described in Barbau-Piednoir et al. (2013a). Only primer pairs that gave in silico the expected amplicon were retained for the following steps.

### Qualitative SYBR® Green qPCR assay and optimal primer concentration

All qPCR assay reactions and analysis of the results were performed according to the protocol described in Barbau-Piednoir et al. (2013a).

The optimal concentration of the selected primer pairs was determined by testing one to three positive isolates with different concentrations of each primer, i.e., between 250 and 1000 nM. The concentration giving the lowest Cq value without formation of a high level of primer dimer was selected. At this selected concentration, a positive sample at a concentration around the limit of detection (LOD) should not present a primer dimer dissociation peak higher than the dissociation peak corresponding to the amplicon derived from the positive sample. The primer pairs used in this study and their optimal concentrations are presented in Table 2.

**Table 2:** Primer pair sequences, concentration, amplicon size, and *T*_*m*_ value for each SYBR® Green qPCR assay

Targeted genus	Targeted pathotype	Targeted gene	Primer pair name	Primer name	Primer sequence, 5′- > 3′	Primer concentration	Product size (bp)	Reference	Average *T*_*m*_ value specificity (°C)
*Escherichia*	All	β-Glucuronidase (*uidA*)	*uidA*-3	*uidA*-3-F-tronc	GCAGTTTCATCAATCACCAC	250 nM	87	This study	78.4
				*uidA*-3-R	CTCCTACCGTACCTCGCATTAC	250 nM			
			*uidA*-7	*uidA*-7-F	GGGATAGTCTGCCAGTTCAGTT	250 nM	83	This study	77.2
				*uidA*-7-R-deg	GATGTCAC***D***CCGTATGTTATTG	250 nM			
*Shigella/ Escherichia*	All/ EIEC	Invasion plasmid antigen H (*ipaH*)	*ipaH*-569	*ipaH*-569-F	CCTTTTCCGCGTTCCTTGA	1000 nM	64	Thiem et al. (2004)	78.5
				*ipaH*-569-R	CGGAATCCGGAGGTATTGC	1000 nM			
			*ipaH*-3	*ipaH*-3-F	ACAGGTGATGCGTGAGACTG	500 nM	101	This study	80.5
				*ipaH*-3-R-deg	ATGAATGGTGCAGT***Y***GTGAG	500 nM			
*Escherichia*	EHEC	Intimin (*eae*)	*eae*-185	*eae*-185-F	CATTGATCAGGATTTTTCTGGTGATA	1000 nM	102	Nielsen et al. (2003)	75.9
				*eae*-185-R	CTCATGCGGAAATAGCCGTTA	1000 nM			
			*eae*-EBP1	*eae*-EBP-1F	CGTCATGGTACGGGTAAT	1000 nM	83	This study	74
				*eae*-EBP-1Rdeg	ATTTGCTGAGACCACGRTTT	1000 nM			
	VTEC	Shiga toxins 1 and 2 (*stx1* & *stx2* (except *2f*))	*stx1&2*–4	*stx1&2*–4-F-deg	TTTGT***Y***ACTGT***S***AC***R***GC***W***GAAGC***Y***TTACG	1000 nM	131–128	Degenerated from Perelle et al. (2004)	*stx1*: 77.2, *stx2:* 78.1
				*stx1&2*–4-R	CCCCAGTTCA***RW***GT***R***AG***R***TCMAC***R***TC	1000 nM		Perelle et al. (2004)	
	*stx1* containing VTEC	Shiga toxins 1 (*stx1*) (all variants)	*stx1*–185	*stx1*–185-F	GTCACAGTAACAAACCGTAACA	250 nM	95	Jothikumar et al. (2002)	77.1
				*stx1*–185-R	TCGTTGACTACTTCTTATCTGGA	250 nM			
	*stx2* containing VTEC	Shiga toxins 2 (*stx2*) (all variants)	*stx2*–81-mix	*stx2*–81-deg-F	GTTTCCATGAC***R***ACGGACAGCAG	250 nM	122	Pavlovic et al. (2010)	78.5 (variant “f”) other variants 79.5–80
				*stx2*–81-alt-R	CTGAACTCCATTAA***MK***CCAGATATG	250 nM		This study	
	Typical EAEC	Transcriptional activator of the aggregative adherence fimbriae (*aggR*)	*aggR*-185	*aggR*-185-F	CAGAATCGTCAGCATCAGCTACA	1000 nM	97	Fukushima et al. (2009)	74.5
				*aggR*-185-R	GATGCCCTGATGATAATATACGGAA	1000 nM			
			*aggR*-2	*aggR*-F2	AACCAGATCCTTATGCAATCAA	500 nM	61	This study	73.5
				*aggR*-R2	ATGAGTTATCAAGCAACAGCAA	500 nM			
	EAEC	*aggR*-Activated island C (*aaiC*)	*aaiC*-EBP1	*aaiC*-EBP1-F	TTATCAGGGGTGTCGTATGC	250 nM	92	This study	74
				*aaiC*-EBP1-Rdeg	CACTCTCTTCTG***S***GGTAAACG	250 nM			
			*aaiC*-EBP2	*aaiC*-EBP2-Ftronc	GCTCTTAGCAGGGAGTTTG	250 nM	113	This study	75
				*aaiC*-EBP2-R	TGAAATGCCTGAGGACAATG	250 nM			

In bold and italics are degenerated nucleotides: D = A or G or T, Y = C or T, S = C or G, R = A or G, W = A or T, M = A or C, K = G or T

### Selectivity test and inclusivity, exclusivity, and accuracy calculation

Primer pairs that passed the in silico evaluation were tested for their selectivity in situ. This selectivity test consisted of two steps:

1. A preliminary selectivity test involving a few target isolates and a few non-target isolates (most important foodborne pathogenic bacteria) was performed. Primer pairs amplifying only the DNA extracted from the target isolates were tested for full selectivity.
2. The full selectivity test allows testing the inclusivity, exclusivity, and accuracy of each developed qPCR assay. This experiment includes target and non-target isolates representing species belonging to 28 genera (76 *E. coli* isolates, 3 other species of the *Escherichia* genus, and 34 isolates from 22 other genera), two mold species and three animal species, and a no-template control (NTC) (Table 1). The non-target microorganisms relevant to test the exclusivity were chosen among taxonomically closely related and not closely related (pathogenic or not) bacteria that can be present in the food matrices (ISO: International Organization for Standardization 2011).

The qPCR reactions were performed with approximately 10^4^ copies of genomic DNA under the conditions referred to above.

Five criteria were set to define a “specific signal” generated in the selectivity of a SYBR® Green qPCR analysis (as described in Barbau-Piednoir 2010). They are (1) the presence or absence of an (exponential) amplification, (2) presence of a single peak upon melting analysis with a unique *T*_*m*_ value, (3) the presence or absence of a single band on agarose gel with (4) a correct size, and (5) the sequence of the amplicon.

The inclusivity, exclusivity, and accuracy of the assay can be calculated from the selectivity test. The inclusivity represents the ability of the assay to detect its targets. The exclusivity represents the ability of the assay to not detect the non-targets. The accuracy represents the closeness of agreement between a test result and the accepted reference value (International Organization for Standardization (ISO) 1993). Their formulas are the following (EU-RL for *E. coli* 2013):

$$ \mathbf{IN}=\frac{\mathbf{TP}}{\left(\mathbf{TP}+\mathbf{FN}\right)}\times \mathbf{100} $$

$$ \mathbf{EX}=\frac{\mathbf{TN}}{\left(\mathbf{TN}+\mathbf{FP}\right)}\times \mathbf{100} $$

$$ \mathbf{AC}=\frac{\left(\mathbf{TP}+\mathbf{TN}\right)}{\mathbf{N}}\times \mathbf{100} $$

where AC is the accuracy, IN is the inclusivity, EX is the exclusivity, TP is the true positive samples, TN is the true negative samples, FP is the false positive samples, FN is the false negative samples, and N is the number of tested samples.

### Dynamic range and calculation of the PCR efficiency

Primer pairs presenting an acceptable selectivity (i.e., amplifying all targets and none of non-target according to the expectation) were subsequently examined for their dynamic range and PCR efficiency as described previously (Barbau-Piednoir et al. 2013a, 2013b). The dynamic range of a qPCR assay is the range of concentrations where it performs linearly. The dynamic range was assessed for the 13 SYBR® Green qPCR of the CoSYPS Path *E. coli* by analyzing in duplicate a serial dilution in a carrier DNA background (4 ng/μL Calf Thymus DNA (CTD) (Invitrogen, Carlsbad, CA, USA)) of pure isolate gDNA (10,000 to 0.01 theoretical genomic copies) of STEC O145:H- (TIAC1681) and STEC O103:H2 (TIAC614) for the *uidA* and *eae* assays, *Shigella flexneri* 2a (12–0081) and *Shigella boydii* 2 (12–0531) for the *ipaH* assays as no EIEC strains were available, STEC O157:H7 (TIAC617) and STEC O118:H16 (TIAC1804) for the *stx1*and*2*–4 assay, STEC O55:H12 (TIAC1703) and STEC O118:H16 (TIAC1804) for the *stx1*–185 assay, STEC O157:H7 (TIAC617) and STEC O145:H28 (TIAC623) for the *stx2*–81 assay, and EAggEC O104:H2 (TIAC2322) and EAggSTEC O104:H4 (TIAC2003) for the *aggR* and *aaiC* assays. The carrier DNA avoids the improper dilution due to low concentration of gDNA. This analysis also allows the assessment of the coefficient of determination (*R*^2^) and the PCR efficiency (*E*) of the SYBR® Green qPCR assays. The coefficient of determination (*R*^2^) is an indicator of the correlation of data regarding the linear regression curve. The PCR efficiency (*E*) can be calculated according to the formula reported by Rutledge and Cote (2003). Although no performance values are given in the last GMO guidelines for qualitative methods (European Network of GMO Laboratories (ENGL) 2015), *R*^2^ ≥ 0.98 and a PCR efficiency ranging between 80 and 120% have been indicated as good performance criteria for the validation of qualitative qPCR methods (Broeders et al. 2014).

### Sensitivity test

The sensitivity of the chromosomal targets (i.e., *uidA*, *eae*, and *aaiC*) was assessed to determine the LOD of these SYBR® Green qPCR assays. The LOD is defined as “the lowest amount or concentration of analyte in a sample,” which can be reliably detected (with a level of confidence of 95%), but not necessarily quantified (ENGL 2015). The strains used were STEC O145:H- (TIAC1681) and STEC O103:H2 (TIAC614) for the *eae* and *uidA* assays and EAggSTEC O104:Hnt (TIAC1951) and EAggSTEC O104:H4 (TIAC2003) for the *aaiC* assays. To determine the LOD, a range of copy numbers between 10 and 0.1 theoretical genomic copies was tested (i.e., 10, 5, 2, 1, 0.5, 0.2, and 0.1). Each dilution was tested in six replicates, for both isolates. Moreover, the analysis was performed at three independent times, under repeatable conditions, resulting in 36 repeats for each dilution point. It has to be noticed that the dilution points beyond the theoretical single genomic copy were carried out to assess the dilution series’ correctness. Indeed, for chromosomal targets, as it is statistically impossible to get amplification in all replicates with the dilution points beyond 1 theoretical genomic copy, none of these dilution points should give 100% of positive signals.

While some of the targets are located on the bacterial chromosome and occur in single copy (i.e., *uidA*, *eae*, and *aaiC*), some other targets are present in several copies. The shiga-toxin genes 1 and 2 (*stx1* and *stx2*) of the STEC strains are prophagic genes, and a single bacterial host can harbor more than one Stx prophage (Fogg et al. 2012). The transcriptional activator of the aggregative adherence fimbriae (*aggR*) of the EAggEC strains is located on a plasmid (Nataro et al. 1994). The invasion plasmid antigen H (*ipaH)* is present in multiple copies both on a plasmid and on the chromosome of EIEC and *Shigella* spp. (Venkatesan et al. 1989). Since these four genes can be present in multiple copies in a single bacterium, the dilution series strategy described above to determine the LOD of the qPCR assays was not performed for these targets. Only the range of genomic copy numbers between 1 and 10 has been tested to confirm that these amounts are detected 100% of the time by these SYBR® Green qPCR assays. The tested strains are STEC O145:H- (TIAC1681 and STEC O103:H2 (TIAC614) for the *stx1* assays, STEC O157:H7 (TIAC617) and STEC O145:H28 (TIAC623) for the *stx2* assays, and EAggSTEC O104:Hnt (TIAC1951) and EAggSTEC O104:H4 (TIAC2003) for the *aggR* assays.

### Repeatability calculation

As described previously (Barbau-Piednoir et al. 2013a, 2013b), to evaluate the repeatability of the assay, independent tests were performed with the same protocol, with the same samples, by the same operator using the same qPCR apparatus within a short interval of time (International Organization for Standardization (ISO) 1993). The repeatability limit (*r*) and the relative standard deviation of repeatability (RSD_r_) were calculated according to ISO 16140:2003 (ISO: International Organization for Standardization 2003). The RSD_r_ should be ≤ 25% for all dilutions above the LOD for quantitative methods, but there is no critical value fixed for RSD_r_ regarding qualitative qPCR methods (ENGL 2015). The RSD_r_ and *r* values of the Cq values were calculated at each dilution point, while the RSD_r_ and *r* values of the *T*_*m*_ values were calculated with all the *T*_*m*_ values coupled with amplification (Cq < 40).

### Reproducibility study and calculation

To evaluate the reproducibility of the assays (International Organization for Standardization (ISO) 1993), independent tests were performed with the same protocol, using the same samples, in two different laboratories, by two different operators using different apparatus, i.e., Bio-Rad iQ5 (Biorad, Hercules, CA) and ABI 7300 (Applied Biosystems, Life Technologies, Foster City, CA). The tested samples consisted of gDNA extracted from STEC, EAggEC, and *Shigella* spp. and subsequently diluted at different concentrations between 10 and 200 genomic copies per reaction. Each sample was analyzed in duplicate by each operator. The positive controls, analyzed in simplicate, used in this analysis are 10^4^ copies/assay of (i) STEC O157:H7 (TIAC 1615) for *uidA*, *eae*, *stx1*, and *stx2*; (ii) *Shigella dysenteriae* 3 (12–1388) for *ipaH* as no EIEC were available; and (iii) EAggEC O104:H2 (TIAC2322) for *aaiC* and *aggR.*

Two reproducibility measures can be calculated from these results: the relative standard deviation of reproducibility (RSD_R_) and the uncertainty (*U*) (Barbau-Piednoir et al. 2013a). The RSD_R_ should be ≤ 35% for all the tested samples (ENGL 2015). The RSD_R_ of the Cq values are calculated for the tested samples. The RSD_R_ of the *T*_*m*_ values are calculated with all the *T*_*m*_ values coupled with amplification (Cq < 40).

### CoSYPS Path *E. coli* on food samples

#### Pathogenic *E. coli* inoculum preparation

STEC O91:H21 (*stx2* positive, ref. TIAC1863), STEC O157:H7 (*eae* and *stx2* positive, ref. TIAC2096), STEC O55:H12 (*stx1* positive, ref. TIAC1873), and STEC O121:H19 (*stx2* and *eae* positive, ref. TIAC1871) were used to artificially contaminate the food samples. To prepare the spike, a single colony was inoculated in 10 ml of BHI broth and cultured at 37 °C without shaking for 16–18 h. This culture was diluted in sterile BHI broth to obtain an OD_600nm_ of 1 (approximately 5.10^8^ CFU/ml). This dilution, called D0, was used as first culture in a 10-fold serial dilution until D-7 in buffered peptone water (BPW). The enumeration of D-6 to D-7 was performed by plating 100 μl of these dilutions in triplicate on nutrient agar plates and incubated for 18 ± 2 h at 37 °C (Table 4). These two dilutions were used to contaminate the food samples.

#### Artificial contamination of food samples

To obtain food samples contaminated with pathogenic *E. coli*, artificial contamination was performed. Salami, tomatoes, red fruits, and minced meat (all free of pathogenic *E. coli* as confirmed by analysis of not artificially contaminated samples (Blank in Table 4)) were purchased at a retail shop. These matrices have been selected as these represent products at risk for pathogenic *E. coli* contamination. Three sub-samples of 25 g of each food sample (matrix) were stomached in 225 ml of buffered peptone water (BPW) medium in a filter stomacher bag. One sub-sample was kept not contaminated (Blank-“matrix name”), and the two others were contaminated with 100 μl of D-6 and D-7 (D-6-matrix name and D-7-matrix name) by adding the bacteria to the already stomached samples (after stomaching to avoid contamination of the lab material) and subsequent soft homogenization through mixing the stomacher bag by hand. Three no matrix controls were also added containing only the 225 ml of BPW, one without contamination (Blank-blank), the two other contaminated as described previously (D-6-Blank and D-7-Blank). Tomatoes were artificially contaminated with STEC O91:H21 (*stx2* positive, ref. TIAC1863), salami was spiked with STEC O157:H7 (*eae* and *stx2* positive, ref. TIAC2096), red fruits were spiked with STEC O55:H12 (*stx1* positive, ref. TIAC1873), and minced meat was spiked with STEC O121:H19 (*stx2* and *eae* positive, ref. TIAC1871).

#### Enrichment step

According to ISO/TS 13136:2012, samples were enriched in 225 ml of BPW for 24 ± 2 h at 37 °C without shaking.

#### DNA extraction from food samples

After 24 h of enrichment, 1 ml of the enriched broth was transferred into a 1.5-ml micro-centrifuge tube, centrifuged for 10 min at 6000×*g* at room temperature, and the supernatant was discarded. DNA was extracted from the pellet with the Nucleospin Food Kit (Macherey-Nagel®, Düren, Germany) according to the manufacturer’s recommendations.

#### CoSYPS Path *E. coli* on food samples

A 1/10 dilution of the DNA extracted from food samples was identified as the best dilution to avoid inhibition of the PCR reaction and to give a positive signal even with low contamination levels of the food matrix. Thus, the dilution 1/10 of the gDNA extract of each sample was analyzed with the 13 qPCR SYBR® Green assays of the CoSYPS Path *E. coli* detection system, with the same PCR program for each assay as detailed in Barbau-Piednoir et al. (2013a) and using the appropriate concentration of each primer (Table 2). The PCR positive controls used in this analysis are 10^4^ genomic copies (GC) of STEC O157:H7 (TIAC 1615) for the *uidA*, *eae*, *stx1*, and *stx2* assays; 10^4^ GC of *S. flexneri* 2a (12–0081) for the *ipaH* assay; and 10^4^ GC of EAggEC O104:H2 (TIAC 2322) for the *aggR* and *aaiC* assays*.* The PCR negative control used in this analysis is a NTC using UltraPure^™^ DNase/RNase-Free Distilled Water (Life Technologies, Foster City, CA, USA) instead of the DNA template.

## Results

### In silico selection of the primer pairs and optimization of primer concentration

As a first step, specific genes to detect and discriminate five pathotypes of *E. coli* were identified.

For the detection of the *E. coli* species, the *uidA* gene was selected as it is present in approximately 97% of *E. coli* isolates (Feng et al. 1991; McDaniels et al. 1996). This gene encodes the β-D-glucuronidase enzyme (Feng et al. 1991; McDaniels et al. 1996). However, this *uidA* gene is also present in approximately 44% of *Shigella* spp., 29% of *Salmonella* spp., and in a few *Yersinia*, *Citrobacter*, *Edwardsiella*, *Hafnia*, *Staphylococcus*, *Streptococcus*, *Corynebacteria*, and *Clostridium* species (Feng et al. 1991; Tryland and Fiksdal 1998). Therefore, as an additional control to discriminate for most *E. coli* and *Shigella*, the *ipaH* gene was chosen for the detection and discrimination of EIEC and *Shigella* isolates, as it is present in all EIEC and *Shigella* but not in other *E. coli* (Ud-Din and Wahid 2014). This gene encodes a type-3 secretion system effector that is involved in the bacteria’s escape from phagosomes of the host cells and in the inhibition of the immune system of the host (Croxen and Finlay 2010; Schroeder and Hilbi 2008). For the detection and discrimination of STEC and EAggSTEC isolates, the *stx1* and *stx2* genes were selected as they encode the two sub-groups of shiga toxins, i.e., Stx1 and Stx2, which are the main virulence factors of STEC (Croxen and Finlay 2010). These toxins suppress an inflammatory response in the host and increase the attachment of the pathogen to the host cell (Croxen and Finlay 2010). Stx2 is more prevalent in hemorrhagic colitis and HUS than Stx1 (Nataro and Kaper 1998). As the *eae* gene is known as the specific marker for EPEC and EHEC strains (Croxen and Finlay 2010), it was picked for the detection and discrimination of EPEC and EHEC isolates. This gene encodes the bacterial outer membrane protein intimin, which is involved in the intimate adherence and effacement of the host cells (Croxen and Finlay 2010). For EAggEC and EAggSTEC isolates, the *aggR* and *aaiC* genes were selected as they are described in literature as the discriminatory genes for these pathotypes (Croxen and Finlay 2010; Dudley et al. 2006). These genes encode, respectively, the transcriptional activator of the aggregative adherence fimbriae and the AggR-activated island C which induces the adherence of EAggEC and EAggSTEC to the intestinal mucosa of the host (Croxen and Finlay 2010; Dudley et al. 2006).

**Fig. 1:**
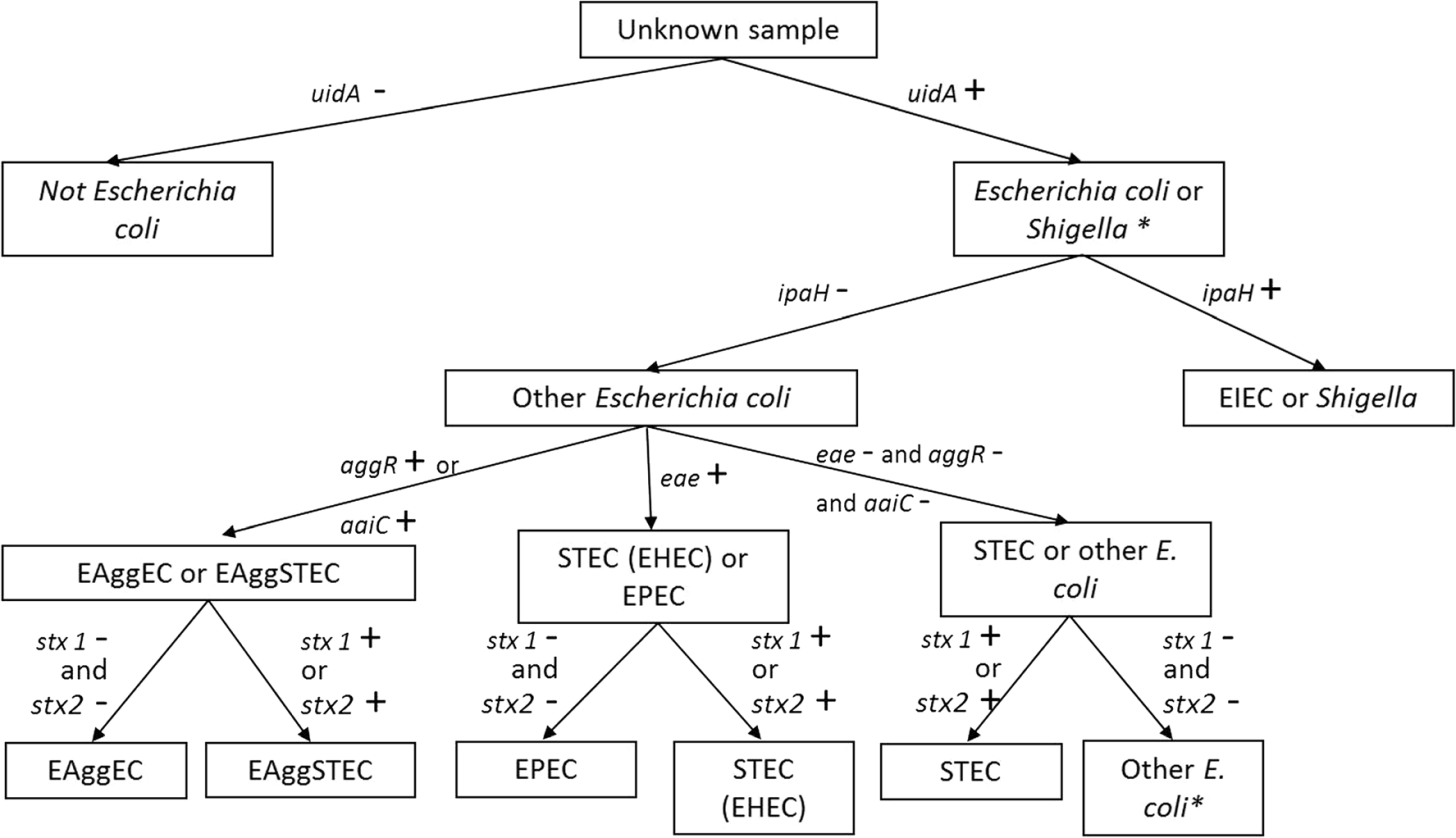
Decision tree of the CoSYPS Path *E. coli*. The CoSYPS Path *E. coli* is a multi-target SYBR® Green qPCR system. Each target is a marker of a pathotype of *E. coli* (except *uidA* which is a marker of *E. coli* (and *Shigella*)). Each level of detection is performed by two SYBR® Green qPCR assays. Abbreviations for the genes are as follows: *uidA* β-D-glucuronidase gene, *ipaH* invasion plasmid antigen H, *aggR* aggregative adherence fimbriae gene, *aaiC aggR*-activated island C, *eae* intimin gene, *stx1* Shiga toxin 1, *stx2* Shiga toxins 2

The primer pairs collected in the literature and those designed during this study (see the “Materials and methods” section) to detect the selected targets were evaluated in silico for their selectivity (data not shown). During this in silico evaluation, some nucleotides were degenerated when necessary. The primer pairs passing the in silico selectivity test were then evaluated in situ. Twenty primer pairs were tested in situ with the preliminary selectivity test (data not shown). From these, 13 primer pairs were retained for 6 targets, with each time two primer pairs for each target in order to avoid/ decrease the risk of false negatives due to mutations in the targeted sequence: *uidA*-3 and *uidA*-7 for *E. coli* detection, *ipaH*-569 and *ipaH*-3 for EIEC and *Shigella* spp. discrimination, *eae*-185 and *eae*-EBP-1 for EPEC and EHEC discrimination, *stx1*and*2*–4, *stx1*–185 and *stx2*–81 for STEC and EASTEC discrimination, and *aggR*-185, *aggR*-2, *aaiC*-EBP1, and *aaiC*-EBP2 for the EAggEC and EAggSTEC discrimination (Fig. 1), when applied to isolates. The optimal concentration of the 13 primer pairs was also evaluated (Table 2). These assays were subsequently experimentally evaluated for their full selectivity.

### Determination of SYBR® Green qPCR assays’ inclusivity, exclusivity, and accuracy calculation

The primer pairs *uidA*-3 and *uidA*-7 amplified 100% of the *E. coli* tested isolates, 83% of the tested *Shigella* spp., and none of the non-target strains or the NTC (Table 1). The primer pairs *ipaH*-569 and *ipaH*-3 and *eae*-185 and *eae*-EBP-1 amplified 100% of their targets *Shigella* strains (no EIEC strains were available in our collection) and *eae*-positive *E. coli* strains and 0% of the non-target strains nor the NTC (Table 1). The *stx1*and *2*–4 assay for STEC detection gave a specific amplification with 90.8% of STEC tested strains and 0% of the non-target strains and NTC (Table 1). The STEC strains not detected by the *stx1*and *2*–4 are all STEC strains containing the variant “f” of the gene *stx2*. In other words, the *stx1*and*2*–4 is able to amplify all variants (a, b, c, d, e, g) of *stx2* (Croxen et al. 2013) except the variant “f.” The *stx1*–185 and *stx2*–81 assays for STEC detection gave a specific amplification with 100% of STEC tested and 0% of the non-target strains and NTC (Table 1). Thus, the *stx2*–81 is amplifying all variants of *stx2* gene including the variant f. The *aggR*-185, *aggR*-2, *aaiC-EBP1*, and *aaiC*-*EBP2* assays for EAggEC and EAggSTEC detection gave a specific amplification with 100% of EAggEC tested (this last result was obtained using a low number of strains (four) due to lack of availability and would require a higher number of strains in order to obtain a more accurate result) and 0% of the non-target strains and NTC (Table 1). Therefore, considering the two assays per target, the detection of each target is 100% accurate.

These 13 assays applied on a positive control showed a unique band at the expected size upon agarose gel analysis (data not shown). Each amplicon was sequenced and shown to correspond to the expected sequence (data not shown). In addition, the 13 detection assays gave a unique melting peak with a specific melting temperature (Table 2).

### Determination of SYBR® Green qPCR assays’ dynamic range and PCR efficiency

The 13 SYBR® Green qPCR assays of the CoSYPS Path *E. coli* applied to isolates performed in a linear manner between 1 and 10,000 copies as their *R*^2^ values were between 0.972 and 0.999 (Table 3). From the dynamic range analyses, the PCR efficiency (*E*) of each assay was calculated. The 13 assays displayed PCR efficiencies ranging between 93.3 and 108.5% (Table 3). Although no performance value are given, in the last GMO guidelines for qualitative methods (ENGL 2015), *R*^2^ ≥ 0.98 and a PCR efficiency ranging between 80 and 120% have been indicated as good performance criteria for the validation of qualitative qPCR methods (Broeders et al. 2014).

**Table 3:** Standard curve, amplification efficiency, coefficient of correlation, LOD, repeatability, and reproductibility of the 13 SYBR® Green qPCR assays for detection and discrimination of pathogenic *E. coli*

				*E. coli* markers		EIEC and *Shigella* markers		EPEC and EHEC markers		STEC and EAggSTEC markers			EAEC and EAggSTEC markers			
			Accepted range	*uidA*-3	*uidA*-7	*ipaH*-569	*ipaH*-3	*eae*-185	*eae*-EBP-1	*stx1*and*2*–4	*stx1*–185	*stx2*–81	*aggR*-185	*aggR*-2	*aaiC*-EBP1	*aaiC*-EBP2
		Standard curve equation	− 3.1 < slope < − 3.6	− 3.1(logX) + 34.7	− 3.2(logX) + 35.1	− 3.3 (logX) + 32.1	− 3.3 (logX) + 32.5	− 3.1(logX) + 34.7	− 3.2(logX) + 35.4	− 3.2(logX) + 36.3	− 3.3(logX) + 35.7	− 3.3 (logX) + 35.3	− 3.3 (logX) + 34.6	− 3.4 (logX) + 35.0	− 3.4(logX) + 35.7	− 3.5 (logX) + 36.2
		Amplification efficiency (E)	89.6–110.2%	108.1%	105.3%	100.1%	99.3%	108.5%	104.0%	106.4%	100.4%	103.0%	100.0%	97.4%	95.3%	93.3%
		Coefficient of correlation (*R*^2^)	> 0.98	0.99	0.99	1.00	1.00	1.00	0.99	0.97 (stx1)/0.98 (stx2)	1.00	0.99	1.00	1.00	0.98	1.00
		LOD (*n* = 36) in copies	≤ 10	2–5	5–10	nt	nt	5–10	2–5	nt	nt	nt	nt	nt	5–10	1–2
		Check LOD (*n* = 12) in copies	≤ 10	nt	nt	<1	<1	nt	nt	5–2	10–5	5–2	5–2	5–2	nt	nt
Repeatability	Isolate 1	LOD Cq RSD_r_ (%)	≤ 25%	2.4	2.5	na	na	2.1	3.5	na	na	na	na	na	1.9	3.1
		LOD Cq r	na	2.2	2.2	na	na	1.9	3.2	na	na	na	na	na	1.7	2.9
		*T*_*m*_ value ± SD (°C)	na	78.2 ± 0.26	76.75 ± 0.26	na	na	75.9 ± 0.21	74.04 ± 0.32	na	na	na	na	na	73.9 ± 0.18	74.7 ± 0.23
		*T*_*m*_ RSD_r_ (%)	≤25%	0.3	0.3	na	na	0.3	0.4	na	na	na	na	na	0.2	0.3
		*T*_*m*_ r	na	0.7	0.7	na	na	0.6	0.9	na	na	na	na	na	0.5	0.7
	Isolate 2	LOD Cq RSD_r_ (%)	≤25%	2.3	2.4	na	na	2.6	2.6	na	na	na	na	na	2.5	3.8
		LOD Cq r	na	2	2.2	na	na	2.3	2.4	na	na	na	na	na	2.2	3.7
		*T*_*m*_ value ± SD (°C)	na	78.2 ± 0.23	77.04 ± 0.16	na	na	75.9 ± 0.23	74.41 ± 0.19	na	na	na	na	na	73.8 ± 0.23	74.7 ± 0.26
		*T*_*m*_ RSD_r_ (%)	≤25%	0.3	0.2	na	na	0.3	0.3	na	na	na	na	na	0.3	0.4
		*T*_*m*_ r	na	0.6	0.4	na	na	0.6	0.5	na	na	na	na	na	0.7	0.7
Reproductibility	Maximum RSD_R_ (%)	Cq	≤ 35%	6.23	6.89	5.24	5.95	2.54	4.36	1.75	3.16	2.72	3.19	3.64	2.58	3.91
		*T*_*m*_	≤35%	0.72	0.87	0.54	0.27	0.7	0.77	0.59	0.46	0.94	0.24	0.15	0.34	0.62
	Uncertainty at 99%	Cq (# positive sample/12 samples)	na	2.96 (12)	3.97 (12)	1.20 (1)	1.37 (1)	1.01 (5)	1.51 (5)	0.92 (6)	1.24 (5)	1.38 (7)	0.96 (1)	1.14 (1)	0.83 (1)	1.23 (1)
		*T*_*m*_ (# positive sample/12 samples)	na	1.09 (12)	1.15 (12)	0.4 (1)	0.2 (1)	0.7 (5)	0.84 (5)	0.59 (6)	0.45 (5)	1.09 (7)	0.17 (1)	0.10 (1)	0.23 (1)	0.44 (1)

Standard curves, *E*, and *R*^2^ were obtained from two replicates for each concentration (from 10,000 copies to 1 copy) from two different strains. LOD of chromosomic targets is based on 18 repetitions of each dilution (10-5-2-1-0.5-0.2-0.1 genomic copies per well) with the two bacterial strains as DNA template (36 repeats in total). Check of LOD is performed for non-chromosomic targets, based on six repeats of dilution points (10-5-2-1 genomic copies per well) with two bacterial strains as DNA template. Repeatability is calculated on the LOD determination test

*X* copy number, *LOD* limit of detection, *na* not applicable, *#* number, *nt* not tested

### Determination of SYBR® Green qPCR assays’ sensitivity and repeatability

The LOD of the chromosomal assays was determined to be between 1 and 10 copies (Table 3 and Supplementary Table S1) complying with the requirement “between 1 and 10 CFU” (ISO: International Organization for Standardization 2011). The *r* values at the LOD of the Cq values ranged between 1.7 and 3.7 Cq and those of the *T*_*m*_ values at all dilutions ranged between 0.4 and 0.9 °C (Table 3). The RSD_r_ values at LOD of the Cq values of the 13 assays were between 1.9 to 3.8% while those of the *T*_*m*_ values at all dilutions ranged between 0.2 to 0.4% (Table 3). The RSD_r_ should be ≤ 25% for all dilutions above the LOD for quantitative methods, but there is no critical value fixed for RSD_r_ regarding qualitative qPCR methods (ENGL 2015).

### Determination of SYBR® Green qPCR assays’ reproducibility

For all the developed SYBR® Green qPCR assays, the RSD_R_ values were below 35% as requested by the ENGL guideline (ENGL 2015), i.e., between 0.09 and 0.94% for the *T*_*m*_ values and between 0.02 and 6.89% for the Cq values (Supplementary Table S2). The uncertainty at 99% of confidence was also calculated from the reproducibility data. *U* was ranging between 0.10 and 1.15 for the *T*_*m*_ values and between 0.83 and 3.97 for the Cq values (Supplementary Table S2).

### CoSYPS Path *E. coli* on food samples

After the validation of the CoSYPS Path *E. coli* system on pure isolates, the performance of the developed SYBR® Green qPCR assays was subsequently tested on real-life food samples. Hereto, different representative food matrices were artificially contaminated at different initial concentrations with different *E. coli* pathotypes, followed by an enrichment step and a total DNA extraction. The negative controls (i.e., non-contaminated food samples) demonstrated absence of natural STEC, EAggEC, EPEC, and EIEC contaminations in the four matrices tested; i.e., *ipaH*, *eae*, *stx1*, *stx2*, *aaiC*, and *aggR* markers were negative in the corresponding qPCR assay (Table 4). Natural presence of *E. coli*, i.e., the *uidA* marker is positive, was shown for minced meat and a weak positive signal was detected in the other food matrices but not in the no-matrix blank (Blank-blank) control. The low level of spiked STEC contamination in each of the four different types of matrices (i.e., tomato, salami, red fruits, and minced meat) was detected after 24-h enrichment using the SYBR® Green qPCR assays of the CoSYPS Path *E. coli* system. Indeed, all expected markers were positive in the qPCR assays using the DNA extracted from the artificially contaminated samples as template (Table 4). The qPCR assays of the CoSYPS Path *E. coli* detection system were able to detect a level of an initial contamination level as low as 2 to 7 cfu/25 g after 24 h of enrichment giving Cq values from 13.4 to 25.5 (Table 4), which are far below the Cq values of the assays at the LOD (i.e., 32.11 to 32.88 (Supplementary Table S1)).

**Table 4:** Detection of spiked pathogenic *E. coli* on four different food matrices using the CoSYPS Path *E. coli* detection system

Sample	Matrix	Spiked strain	Serotype	Expected positives	Inoculum cfu/25 g	qPCR assay of the CoSYPS Path *E. coli*												
						*uidA*-3	*uidA*-7	*ipaH*-569	*ipaH*-3	*eae*-185	*eae*-EBP1	*stx1*and*2*–4	*stx1*–185	*stx2*–81-alt	*aggR*-185	*aggR*-2	*aaiC*-EBP1	*aaiC*-EBP2
Positive control	na	na	na	all	na	+	+	+	+	+	+	+	+	+	+	+	+	+
No-template control	na	None	na	None	na	–	–	–	–	–	–	–	–	–	–	–	–	–
D-6-Tomato	Tomato	TIAC 1863	O91:H21	*stx2*	48 ± 4	+(13.7)	+(13.7)	–	–	- *	–	+(14.7)	–	+(13.5)	–	–	- *	–
D-7-Tomato					5 ± 1	+(15.5)	+(15.0)	–	–	–	–	+(16.8)	–	+(15.2)	–	–	–	–
Blank-Tomato					None	- (33.2)	- (34.4)*	–	–	–	–	–	–	- *	–	–	–	–
D-6-Salami	Salami	TIAC 2096	O157:H7	*eae*/*stx2*	27 ± 15	+(18.1)	+(18.6)	–	–	+(18.7)	+(19.2)	+(18.8)	–	+(17.0)	–	–	–	–
D-7-Salami					3 ± 1	+(18.4)	+(18.8)	–	–	+(18.5)	+(19.8)	+(18.1)	–	+(16.5)	–	–	–	–
Blank-Salami					None	- *	- *	–	–	–	–	–	–	–	–	–	–	–
D-6-Red fruits	Red fruits	TIAC 1873	O55:H12	*stx1*	56 ± 17	+(19.3)	+(19.2)	–	–	–	–	+(23.0)	18.8	- *	–	- *	–	–
D-7-Red fruits					± 7	+(20.7)	+(20.7)	–	–	–	–	+(22.7)	19.2	–	–	–	–	–
Blank-Red fruits					None	- *	- *	–	–	–	–	–	–	–	–	–	–	–
D-6-minced meat	Minced meat	TIAC 1871	O121:H19	*eae*/*stx2*	64 ± 13	+(15.4)	+(15.3)	–	- *	+(17.9)	+(18.2)	+(18.7)	–	+(17.2)	–	–	–	–
D-7-minced meat					4 ± 2	+(14.8)	+(15.2)	–	–	+(24.5)	+(24.6)	+(25.5)	–	+(23.5)	–	–	–	–
Blank minced meat					None	+(15.4)	+(14.9)	–	–	–	–	–	–	–	–	–	–	–
D-6-Blank	None	TIAC 2096	O157:H7	*eae*/*stx2*	27 ± 15	+(14.7)	+(14.8)	–	–	+(14.9)	+(14.9)	+(15.0)	–	+(13.4)	–	–	–	–
D-7-Blank					3 ± 1	+(14.9)	+(14.4)	-*	–	+(14.6)	+(16.0)	+(14.8)	–	+(13.4)	–	–	–	–
Blank-blank					None	–	–	–	–	–	–	–	–	–	–	–	–	–

*+* there is specific amplification (with the correct *T*_*m*_ value), *−* no amplification, −* weak signal probably due to low contamination level was observed, *Cq > 32 and < LOD* Cq value observed with Cq > LOD, *na* not applicable

## Discussion

In the EU, STEC is the fourth cause of human zoonosis, particularly the O157 serogroup (European Food Safety Authority (EFSA), European Centre for Disease Prevention and Control (ECDC) Prevention and Control, 2016). Until 2013, the reference method for the detection of STEC (ISO 16654:2001 2001) was limited to the detection of *E. coli* O157. This method does, however, not include virulence gene detection. To deal with the detection of the new O104:H4 serotype (King et al. 2012; Wadl et al. 2011) causing the large German and French *E. coli* outbreak, the EU has adapted and extended its related regulation and a new standard for the detection of these serogroups was published. In this new standard, prior to detection of the serogroup by qPCR, a sample is screened by qPCR for the presence of the main virulence factors of STEC, i.e., *stx1*, *stx2* (able to be transferred to other serogroups as proven by the 2011 outbreak), and *eae*. This is a significant change in the detection strategy of STEC compared to the previous *E. coli* detection method (ISO 16654:^2001^), where only serogroup O157 was targeted without any consideration of the presence of particular virulence genes.

In line with this new STEC screening strategy, the inclusion of other virulence factors from *E. coli* pathotypes other than STEC, such as EAggEC, EAggSTEC, EIEC, and EPEC, could be considered in order to better evaluate the possible presence of pathogenic *E. coli* in a food sample.

In this study, the combinatory SYBR® Green qPCR screening system for pathogenic *E. coli* (CoSYPS Path *E. coli*) was developed and validated on isolates and explored on food samples in order to answer this need. Although next-generation sequencing (NGS) is now becoming a standard for surveillance and typing of bacterial isolates (whole-genome sequencing), metagenomics on more complex samples is still far from becoming routine practice as it remains too expensive and rather sophisticated in data analysis as compared to CoSYPS Path *E. coli* for rapid screening of pathogens in food samples. The 13 SYBR® Green qPCR assays developed and validated in this study allow the detection of six genes of interest allowing the discrimination of *E. coli*, *Shigella*, and five pathotypes of *E. coli* isolates (Fig. 1). It has to be noticed that if applied to food samples, the *uidA* gene can also be positive if *Salmonella* is present in the tested sample (Feng et al. 1991; Tryland and Fiksdal 1998). Thus, if *uidA* is the only positive gene, the CoSYPS *Salmonella* (Barbau-Piednoir et al. 2013b) could be run to check for the presence of *Salmonella* in the sample. Two SYBR® Green qPCR assays have been developed for each targeted gene in order to avoid false negatives due to polymorphisms in the primer annealing sites and for detection of all variants of the targeted gene. The 13 assays have been tested for their exclusivity, inclusivity, and accuracy. All of them indicate, using the number of strains available for the test, an efficient detection of the target with an accuracy of 100%, except for the assay *stx1*and*2*–4 which does not detect the variant f of the *stx2* gene (accuracy at 90.5%; Table 1). This variant, first described in pigeon isolates, was rarely associated with symptomatic human infections and was therefore not included in ISO /TS 13136:2012. However, recently, an increase of *stx2f* variants was observed in human isolates in the Netherlands and this was linked to mild disease (Friesema et al. 2014) and occasionally severe disease (Friesema et al. 2015). The second assay of the *stx2* gene in the CoSYPS Path *E. coli* system, i.e., *stx*2–81, detects all variants of the *stx2* gene including the variant f. Thus, with the two assays, all variants of *stx2* gene are detected. This is an added value of the CoSYPS Path *E. coli* system presented in this paper. Therefore, considering both assays of each target, the detection of the seven targets (six virulence genes and one *E. coli* marker) of the CoSYPS Path *E. coli* is 100% accurate for all the strains tested in this assay. The LOD of the chromosomal assay has been studied and is between 1 to 10 genomic copies, which complies with the foodborne PCR performance requirements of ISO 22118:2011 (2011). The detection at these levels was also confirmed for non-chromosomal assays. The dynamic range, PCR efficiency, repeatability, and reproducibility of each developed assay were also evaluated and compared with the European requirements for qPCR detection assays for GMO detection (ENGL 2015), where qPCR is the gold standard for detection. All these parameters complied with the EU requirements for the developed assays except for the *R*^2^ of the *stx1* and *2*–4, which is below the required 0.98. This is of low importance as the assays are used qualitatively in the CoSYPS Path *E. coli* system. In conclusion, this validation demonstrates that the SYBR® Green qPCR methods developed in this study are compliant with the requirements of an efficient qPCR assay. In addition to the above mentioned advantages of being cheap and allowing melting-curve analysis, the CoSYPS Path *E. coli* system has other benefits. First, the CoSYPS Path *E. coli* system is homogeneous; i.e., all SYBR® Green qPCR assays can be run on a single 96-well plate as they are all validated with the same PCR program. Secondly, each SYBR® Green qPCR assay constituting the CoSYPS Path *E. coli* system shows a satisfactory inclusivity, exclusivity, repeatability, and reproducibility, as demonstrated during its validation. Thirdly, the CoSYPS Path *E. coli* analysis is fast. Indeed, the screening results can be obtained 1 day after receiving the suspected food samples, which already indicates the possible presence of one of the five pathotypes. Further analyses including the isolation of the strain and subsequent confirmation of the pathotype will however be needed, as a final conclusion requires the presence of the detected target genes within one genome (isolate). Fourthly, due to its modularity, in case of appearance of a new emerging hybrid strain as observed in the O104:H4 outbreak (King et al. 2012; Wadl et al. 2011), new targets can be easily and rapidly added to the existing CoSYPS Path *E. coli* system. Last but not least, the CoSYPS Path *E. coli* could be combined with the 11 SYBR® Green qPCR assays previously developed and validated for the *Listeria* (CoSYPS *Listeria*) and *Salmonella* (CoSYPS *Salmonella*) detection and discrimination (Barbau-Piednoir et al. 2013a, 2013b, 2015). These 24 assays constitute a multi-pathogen screening system, which is called CoSYPS Path Food system for “Combinatory SYBR® Green qPCR Screening system for pathogen detection in food samples.” As for the individual species-specific CoSYPS systems, this screening system could be run in a single 96-well plate as all developed qPCR assays use the same PCR program. Furthermore, since also this approach is modular, selected assays could be run individually or more qPCR assays could be combined to detect a wider range of foodborne pathogens or emerging pathogens in a same sample. The only requirement to add a new assay in this modular CoSYPS Path Food system is to develop an assay that is able to be run under the same conditions, allowing its use in high-throughput modus in the same 96-well plate. Additionally, to allow a user-friendly and automated data analysis of the CoSYPS Path Food results, especially when such a large number of qPCR assays are run, a decision support system (DSS) has been previously developed (Van den Bulcke et al. 2010). Combined with this DSS, the CoSYPS Path Food detection system offers a very useful approach for a high-quality screening for food samples, which makes it a remarkable food surveillance tool which can be modulated in response to the laboratory needs. It will also considerably reduce the time and the cost of a sample analysis. Such simultaneous detection may be useful when a global screening and rapid identification of foodborne pathogens is requested, as in the case of a bio-emergency or outbreak of unknown origin.

The present paper focuses on the targeted genes and the performance criteria of the qPCR method. The method is fully validated for the use with isolates. As a proof of concept, the CoSYPS Path Food screening system was tested on four artificially contaminated representative food matrices. For full implementation of the workflow for food samples, it is recommended to extend the number of tested food matrices, and also to include sprouts, sprouted seeds, and the irrigation water obtained during the sprouting process for which a legislation exists, and to test more strains per matrix. This would confirm the full applicability of the system for food in the context of the EU legislation and ISO norms currently used by the EU enforcement laboratories.

## Electronic supplementary material

Supplementary Table S1 (XLSX 38 kb)

## References

[CR1] Anklam KS, Kanankege KS, Gonzales TK, Kaspar CW, Dopfer D (2012). Rapid and reliable detection of Shiga toxin-producing *Escherichia coli* by real-time multiplex PCR. J Food Prot.

[CR2] Aranda KR, Fagundes-Neto U, Scaletsky IC (2004). Evaluation of multiplex PCRs for diagnosis of infection with diarrheagenic *Escherichia coli* and *Shigella* spp. J Clin Microbiol.

[CR3] Baccin Fialho O, Maltempi de Souza E, de Borda DC, de Oliveira PF, Klassen G, Irino K, Paludo KS, Araujo de Assis FE, Surek M, de Souza Santos Farah SM, Telless Fadel-Picheth CM (2013). Detection of diarrheagenic *Escherchia coli* using a two-system multiplex-PCR protocol. J Clin Lab Anal.

[CR4] Barbau-Piednoir E, Botteldoorn N, Mahillon J, Dierick K, Roosens NH (2015). Fast and discriminative CoSYPS detection system of viable *Salmonella* spp. and *Listeria* spp. in carcass swab samples. Int J Food Microbiol.

[CR5] Barbau-Piednoir E, Botteldoorn N, Yde M, Mahillon J, Roosens NH (2013). Development and validation of qualitative SYBR® Green real-time PCR for detection and discrimination of *Listeria* spp. and *Listeria monocytogenes*. Appl Microbiol Biotechnol.

[CR6] Barbau-Piednoir E, Roosens NH, Bertrand S, Mahillon J, Botteldoorn N (2013). SYBR® Green qPCR *Salmonella* detection system allowing discrimination at the genus, species and subspecies levels. Appl Microbiol Biotechnol.

[CR7] Barletta F, Ochoa TJ, Cleary TG (2013). Multiplex real-time PCR (MRT-PCR) for diarrheagenic. Methods Mol Biol.

[CR8] Botteldoorn N, Heyndrickx M, Rijpens N, Herman L (2003). Detection and characterization of verotoxigenic *Escherichia coli* by a VTEC/EHEC multiplex PCR in porcine faeces and pig carcass swabs. Res Microbiol.

[CR9] Broeders S, Huber I, Grohmann L, Berben G, Taverniers I, Mazzara M, Roosens N, Morisset D (2014). Guidelines for validation of qualitative real-time PCR methods. Tr food. Sci Technol.

[CR10] Bugarel M, Beutin L, Fach P (2010). Low-density macroarray targeting non-locus of enterocyte effacement effectors (*nle* genes) and major virulence factors of Shiga toxin-producing *Escherichia coli* (STEC): a new approach for molecular risk assessment of STEC isolates. Appl Environ Microbiol.

[CR11] Chandra M, Cheng P, Rondeau G, Porwollik S, McClelland M (2013). A single step multiplex PCR for identification of six diarrheagenic *E. coli* pathotypes and *Salmonella*. Int J Med Microbiol.

[CR12] Clements A, Young JC, Constantinou N, Frankel G (2012). Infection strategies of enteric pathogenic *Escherichia coli*. Gut Microbes.

[CR13] Commission of the European Communities (2005) Commission regulation (EC) no 2073/2005 of 15 November 2005 on microbiological criteria for foodstuffs. Off J Eur Union L338

[CR14] Commission of the European Communities (2013) Commission regulation (EU) no 209/2013 of 11 march 2013 amending regulation (EC) no 2073/2005 as regards microbiological criteria for sprouts and the sampling rules for poultry carcases and fresh poultry meat. Off J Eur Union L68

[CR15] Croxen MA, Finlay BB (2010). Molecular mechanisms of *Escherichia coli* pathogenicity. Nat Rev Microbiol.

[CR16] Croxen MA, Law RJ, Scholz R, Keeney KM, Wlodarska M, Finlay BB (2013). Recent advances in understanding enteric pathogenic *Escherichia coli*. Clin Microbiol Rev.

[CR17] Dudley EG, Thomson NR, Parkhill J, Morin NP, Nataro JP (2006). Proteomic and microarray characterization of the AggR regulon identifies a *pheU* pathogenicity island in enteroaggregative *Escherichia coli*. Mol Microbiol.

[CR18] EU-RL for *E. coli* (2013) CEN ISO/TS 13136:2012-Report on the primary validation of the PCR screening reactions and the determination of the performance parameters, based on the results of the analytical tests carried out within the EU-RL VTEC proficiency testing program (2009–2012) EU Reference Laboratory for *E. coli*, Istituto Superiore di Sanità, Roma, Italy

[CR19] European Food Safety Authority (EFSA), European Centre for Disease Prevention and Control (ECDC) (2013). The European Union summary report on trends and sources of zoonoses, zoonotic agents and food-borne outbreaks in 2011. EFSA J.

[CR20] European Food Safety Authority (EFSA), European Centre for Disease Prevention and Control (ECDC) Prevention and Control (2016). The European Union summary report on trends and sources of zoonoses, zoonotic agents and food-borne outbreaks in 2015. EFSA J.

[CR21] EFSA (European Food Safety Authority) and ECDC (European Centre for Disease Prevention and Control) (2015). The European Union summary report on trends and sources of zoonoses, zoonotic agents and food-borne outbreaks in 2014. EFSA J.

[CR22] European Network of GMO Laboratories (ENGL) (2015). Definition of minimum performance requirements for analyticals methods of GMO testing.

[CR23] Feng P, Lum R, Chang GW (1991). Identification of *uidA* gene sequences in β-D-glucuronidase-negative *Escherichia coli*. Appl Environ Microbiol.

[CR24] Fogg PC, Saunders JR, McCarthy AJ, Allison HE (2012). Cumulative effect of prophage burden on Shiga toxin production in *Escherichia coli*. Microbiology.

[CR25] Friesema I, van der Zwaluw K, Schuurman T, Kooistra-Smid M, Franz E, van Duynhoven Y, van Pelt W (2014). Emergence of *Escherichia coli* encoding Shiga toxin *2f* in human Shiga toxin-producing *E. coli* (STEC) infections in the Netherlands, January 2008 to December 2011. Euro Surveill.

[CR26] Friesema IH, Keijzer-Veen MG, Koppejan M, Schipper HS, van Griethuysen AJ, Heck ME, van Pelt W (2015). Hemolytic uremic syndrome associated with *Escherichia coli* O8:H19 and Shiga toxin *2f* gene. Emerg Infect Dis.

[CR27] Fukushima H, Katsube K, Tsunomori Y, Kishi R, Atsuta J, Akiba Y (2009). Comprehensive and rapid real-time PCR analysis of 21 foodborne outbreaks. Int J Microbiol.

[CR28] International Organization for Standardization (ISO) (1993). ISO 3534–1:1993 statistics—vocabulary and symbols—part 1.

[CR29] ISO: International Organization for Standardization (2001). ISO 16654:2001 Microbiologie des aliments—méthode horizontale pour la recherche des *Escherichia coli* O157.

[CR30] ISO: International Organization for Standardization (2003) ISO 16140:2003-microbiology of food and animal feeding stuffs—protocol for the validation of alternative methods. International Organization for Standardization, Geneva

[CR31] ISO: International Organization for Standardization (2011) ISO 22118:2011-microbiology of food and animal feeding stuffs— polymerase chain reaction (PCR) for the detection and quantification of food-borne pathogens—performance characteristics of molecular detection methods. International Organization for Standardization, Geneva

[CR32] ISO: International Organization for Standardization (2012) ISO/TS 13136:2012 microbiology of food and animal feed - real-time polymerase chain reaction (PCR)-based method for the detection of food-borne pathogens - horizontal method for the detection of shiga toxin-producing *Escherichia coli* (STEC) and the determination of O157, O111, O26, O103 and O145 serogroups. International Organization for Standardization, Geneva

[CR33] Karmali MA, Gannon V, Sargeant JM (2010). Verocytotoxin-producing *Escherichia coli* (VTEC). Vet Microbiol.

[CR34] Kim IW, Kang MH, Kwon SH, Cho SH, Yoo BS, Han SH, Yoon BS (2010). Rapid detection of virulence *stx2* gene of enterohemorrhagic *Escherichia coli* using two-step ultra-rapid real-time PCR. Biotechnol Lett.

[CR35] King LA, Nogareda F, Weill FX, Mariani-Kurkdjian P, Loukiadis E, Gault G, Jourdan-DaSilva N, Bingen E, Mace M, Thevenot D, Ong N, Castor C, Noel H, Van CD, Charron M, Vaillant V, Aldabe B, Goulet V, Delmas G, Couturier E, Le SY, Combe C, Delmas Y, Terrier F, Vendrely B, Rolland P, de Valk H (2012). Outbreak of Shiga toxin-producing *Escherichia coli* O104:H4 associated with organic fenugreek sprouts, France, June 2011. Clin Infect Dis.

[CR36] Kuwayama M, Shigemoto N, Oohara S, Tanizawa Y, Yamada H, Takeda Y, Matsuo T, Fukuda S (2011). Simultaneous detection of virulence factors from a colony in diarrheagenic *Escherichia coli* by a multiplex PCR assay with Alexa Fluor-labeled primers. J Microbiol Methods.

[CR37] Liu J, Gratz J, Amour C, Kibiki G, Becker S, Janaki L, Verweij JJ, Taniuchi M, Sobuz SU, Haque R, Haverstick DM, Houpt ER (2013). A laboratory-developed TaqMan® array card for simultaneous detection of 19 enteropathogens. J Clin Microbiol.

[CR38] McDaniels AE, Rice EW, Reyes AL, Johnson CH, Haugland RA, Stelma GN (1996). Confirmational identification of *Escherichia coli*, a comparison of genotypic and phenotypic assays for glutamate decarboxylase and β-D-glucuronidase. Appl Environ Microbiol.

[CR39] Nataro JP, Kaper JB (1998). Diarrheagenic *Escherichia coli*. Clin Microbiol Rev.

[CR40] Nataro JP, Yikang D, Yingkang D, Walker K (1994). AggR, a transcriptional activator of aggregative adherence fimbria I expression in enteroaggregative *Escherichia coli*. J Bacteriol.

[CR41] Nielsen EM, Andersen MT (2003). Detection and characterization of verocytotoxin-producing by automated 5′ nuclease PCR assay. J Clin Microbiol.

[CR42] Paton AW, Paton JC (1998). Detection and characterization of Shiga toxigenic *Escherichia coli* by using multiplex PCR assays for *stx1, stx2*, *eaeA*, enterohemorrhagic *E. coli hlyA, rfbO111*, and *rfbO157*. J Clin Microbiol.

[CR43] Pavlovic M, Huber I, Skala H, Konrad R, Schmidt H, Sing A, Busch U (2010). Development of a multiplex real-time polymerase chain reaction for simultaneous detection of enterohemorrhagic *Escherichia coli* and enteropathogenic *Escherichia coli s*trains. Foodborne Pathog Dis.

[CR44] Perelle S, Dilasser F, Grout J, Fach P (2004). Detection by 5′-nuclease PCR of Shiga-toxin producing *Escherichia coli* O26, O55, O91, O103, O111, O113, O145 and O157:H7, associated with the world’s most frequent clinical cases. Mol Cell Probes.

[CR45] Rozen S, Skaletsky H (2000). Primer 3 on the WWW for general users and for biologist programmers. Methods Mol Biol.

[CR46] Rutledge RG, Cote C (2003). Mathematics of quantitative kinetic PCR and the application of standard curves. Nucleic Acids Res.

[CR47] Ryan KJ (2004) Enterobacteriaceae. In: Ryan KJ, Ray CG (eds) An introduction to infectious diseases. Sherris medical microbiology, McGraw-hill, New York, pp 343–371. 10.1036/0838585299

[CR48] Scheutz F, Nielsen EM, Frimodt-Moller J, Boisen N, Morabito S, Tozzoli R, Nataro JP, Caprioli A (2011). Characteristics of the enteroaggregative Shiga toxin/verotoxin-producing *Escherichia coli* O104:H4 strain causing the outbreak of haemolytic uraemic syndrome in Germany, may to June 2011. Euro Surveill.

[CR49] Schroeder GN, Hilbi H (2008). Molecular pathogenesis of *Shigella* spp.: controlling host cell signaling, invasion, and death by type III secretion. Clin Microbiol Rev.

[CR50] Sharma VK, Dean-Nystrom EA, Casey TA (1999). Semi-automated fluorogenic PCR assays (TaqMan®) for rapid detection of *Escherichia coli* O157:H7 and other shiga toxigenic *E. coli*. Mol Cell Probes.

[CR51] Sharma VK, Dean-Nystrom EA (2003). Detection of enterohemorrhagic *Escherichia coli* O157:H7 by using a multiplex real-time PCR assay for genes encoding intimin and Shiga toxins. Vet Microbiol.

[CR52] Takahashi H, Kimura B, Tanaka Y, Shinozaki J, Suda T, Fujii T (2009). Real-time PCR and enrichment culture for sensitive detection and enumeration of *Escherichia coli*. J Microbiol Methods.

[CR53] Thiem VD, Sethabutr O, von SL, Tran VT, Do GC, Bui TC, Le HT, Lee H, Houng HS, Hale TL, Clemens JD, Mason C, Dang DT (2004). Detection of *Shigella* by a PCR assay targeting the *ipaH* gene suggests increased prevalence of shigellosis in Nha Trang, Vietnam. J Clin Microbiol.

[CR54] Tryland I, Fiksdal L (1998). Enzyme characteristics of β-D-galactosidase- and β-D-glucuronidase-positive bacteria and their interference in rapid methods for detection of waterborne coliforms and *Escherichia coli*. Appl Environ Microbiol.

[CR55] Tzschoppe M, Martin A, Beutin L (2012). A rapid procedure for the detection and isolation of enterohaemorrhagic *Escherichia coli* (EHEC) serogroup O26, O103, O111, O118, O121, O145 and O157 strains and the aggregative EHEC O104:H4 strain from ready-to-eat vegetables. Int J Food Microbiol.

[CR56] Ud-Din A, Wahid S (2014). Relationship among *Shigella* spp. and enteroinvasive *Escherichia coli* (EIEC) and their differentiation. Braz. J Microbiol.

[CR57] Van den Bulcke M, Lievens A, Barbau-Piednoir E, Mbongolombella G, Roosens N, Sneyers M, Leunda-Casi A (2010). A theoretical introduction to "combinatory SYBR® Green qPCR screening", a matrix-based approach for the detection of materials derived from genetically modified plants. Anal Bioanal Chem.

[CR58] Venkatesan MM, Buysse JM, Kopecko DJ (1989). Use of *Shigella flexneri ipaC* and *ipaH* gene sequences for the general identification of *Shigella* spp. and enteroinvasive *Escherichia coli*. J Clin Microbiol.

[CR59] Wadl M, Rieck T, Nachtnebel M, Greutelaers B, An der Heiden M, Altmann D, Hellenbrand W, Faber M, Frank C, Schweickert B, Krause G, Benzler J, Eckmanns T (2011). Enhanced surveillance during a large outbreak of bloody diarrhoea and haemolytic uraemic syndrome caused by Shiga toxin/verotoxin-producing *Escherichia coli* in Germany, May to June 2011. Euro Surveill.

[CR60] Wasilenko JL, Fratamico PM, Sommers C, DeMarco DR, Varkey S, Rhoden K, Tice G (2014). Detection of Shiga toxin-producing *Escherichia coli* (STEC) O157:H7, O26, O45, O103, O111, O121, and O145, and *Salmonella* in retail raw ground beef using the DuPont BAX(R) system. Front Cell Infect Microbiol.

